# “There must be Someone’s Name Under Every Bit of Text, Even if it is Unimportant or Incorrect”: Plagiarism as a Learning Strategy

**DOI:** 10.1007/s10805-021-09419-z

**Published:** 2021-06-17

**Authors:** Beata Bielska, Mateusz Rutkowski

**Affiliations:** Institute of Sociology, Nicolaus Copernicus University in Torun, Torun, Poland

**Keywords:** Academic integrity, Academic misconduct, Plagiarism, Contract cheating, Poland

## Abstract

The article offers analyses of the phenomenon of copying (plagiarism) in higher education. The analyses were based on a quantitative survey using questionnaires, conducted in 2019 at one of the Polish universities. Plagiarism is discussed here both as an element of the learning process and a subject of public practices. The article presents students’ definitions of plagiarism, their strategies for unclear or difficult situations, their experiences with plagiarism and their opinions on how serious and widespread this phenomenon is. Focusing on the non-plagiarism norm, that is the rule that students are not allowed to plagiarize, and in order to redefine it we have determined two strategies adopted by students. The first is withdrawing in fear of making a mistake (omitting the norm), which means not using referencing in unclear situations, e.g. when the data about the source of information are absent. The second is reducing the scope of the norm applicability (limiting the norm), characterized by the fact that there are areas where the non-plagiarism norm must be observed more closely and those where it is not so important, e.g. respondents classify works as credit-level and diploma-level texts, as in the credit-level work they “can” sometimes plagiarize since the detection rate is poor and consequences are not severe. The presented results are particularly significant for interpreting plagiarism in an international context (no uniform definition of plagiarism) and for policies aimed at limiting the scale of the phenomenon (plagiarism detection systems^1^).

## Introduction

Dynamic transformations in higher education, observed in Western Europe and the USA since the 1960s, in former Eastern Bloc countries after 1989, and a practically global phenomenon today (Antonowicz, 2015a, p. 65–67; Doroholschi et al., 2018, p. 5–6; Kwiek, 2015, p. 52–57; Marginson & van der Wende, 2007; Teichler, 2019, p. 5), not only satisfy the educational and professional aspirations of graduates and their families, but also quite frequently lead to unintended consequences (Boudon, 2008). One of them is devaluation of higher education associated with growing plagiarism among students. There is a common pursuit of education and academic qualifications, which is a result of the popularization of the concept of welfare state, as well as of transformations towards knowledge-based economy (Collins, 1979; Pinheiro & Antonowicz, 2014, p. 299). Simultaneously, studying has been increasingly commercialized – especially after the 2008 economic crisis when many governments decided to reduce the public spending on research and higher education (Courtois & O’Keefe, 2015). The same could be expected after the Covid-19 crisis (Derrick, 2020).

In Poland specifically, private tertiary schools proliferated (and then, like in other post-communist countries, they started to disappear – Kwiek, 2015, p. 107–145). Both public and private schools began to offer paid studies on a wide scale.^1^ The teaching load of academic teachers was increasing, particularly among teachers of the humanities and social sciences; there was also a period when the generation of the 1980s demographic boom finished secondary education (Antonowicz, 2015b, p. 154–155; Rozmus & Kurek-Ochmańska, 2015). Universities started to be included in international rankings, a factor more and more frequently used in public discourse to evaluate them (cf. an interesting discussion about rankings measuring equity in Pitman et al., 2020). A higher education diploma still does not guarantee a good job, but it increases the chance of earning a higher income – though its significance is decreasing in the latter case (Bielska, 2015, p. 50, Polish Central Statistical Office [GUS], 2014, p. 64; GUS, 2016, p. 53). It can also influence the social status of the student and their family (Czarnecki, 2015; Kopycka, 2021; Kwiek, 2015, p. 79–106; cf. category of educational failures and successes in: Smużewska et al., 2015). Although in the last years the number of students at universities in Poland has clearly decreased (GUS, 2019, p. 1), the incentives to bypass official education paths during studies have not disappeared. In such circumstances students can employ strategies for cheating and plagiarism, becoming part of the student culture of copying, whose development is one of the causes and indicators of a decrease in the quality of education.

*Student culture of copying* is understood by us as “perpetuated and transmitted values, norms, attitudes and behavioural patterns of students, related to permanent and common acceptance of breaking the official norms regarding the fulfilment of the social role of a student” (Bielska, 2015, p. 19), and the copying is operationalized (in a narrower meaning) as ways of obtaining credits for academic courses in ways which are non-compliant with law or academic rules (study regulations, codes of ethics, the Penal Code, the Act on Copyrights and Related Rights) (cf. Bielska & Hoffman, 2013, p. 4). Considered in the simplest way, this means cheating and plagiarism. For the purpose of this article, let us focus on *plagiarism*, that is, ascribing the authorship of another person’s work to oneself (e.g. Helgesson & Eriksson, 2014, p. 91–93; Shahabuddin, 2009, p. 353–354). However, it should be borne in mind that plagiarism – understood as theft of intellectual property—is a non-universal cultural construct. It is inextricably linked to the idea of copyright, socially constructed during the late eighteenth century, considered in the Western world as the Romantic era (Angélil-Carter, 2014, p. 15–21; Kobus, 2018; Scollon, 1995). We should also distinguish plagiarism from unethical authorship. The latter is associated either with an inclusion of persons who do not meet the criteria for authorship, or, conversely, with the concealment of the real performers of scientific work (Gureev et al., 2019; Olesen et al., 2018). In our article unethical authorship is a form of plagiarism.

From the sociological perspective used here, social norm is understood as a commonly accepted, either official or unofficial, rule of behaviour. Norms inform what should or should not be done under specific circumstances. They are accompanied by positive and negative sanctions – “rewards” and “punishments”. In this article “the non-plagiarism norm” is the rule according to which students are not allowed to plagiarize. The official norms are usually written and publicly announced, while the unofficial ones are rather “common sense”.

The primary aim of the article is to analyse the perception of plagiarism among students. We have chosen to analyse plagiarism for several reasons. Firstly, although both phenomena (cheating and plagiarism) involve copying^2^ somebody else’s work and thus they have been researched together, the Polish regulations pertaining to them differ: plagiarism may be punished more severely. Secondly, earlier research (Bielska & Hoffman, 2013, p. 48-51) demonstrated that they are perceived by students differently: copying someone’s work is considered to be more common and less blameworthy, while plagiarism is seen as more reprehensible and its scale of occurrence is hardly known. Hence, we are discussing the phenomena governed by different social norms and related to slightly different aspects of the culture of studying.

## Copying in Higher Education

Research on student culture of copying belongs to the field of interdisciplinary studies on higher education (Antonowicz, 2015a, p. 21–67; Kwiek, 2015, p. 52), and our study falls particularly within the field of sociology of higher education in its aspect of educational research. Therefore, we are interested in students plagiarism, not faculty plagiarism, although we are aware that these two are interconnected. Indiscriminate access and commercialization of higher education created conditions fostering the development of student culture of copying. Consequently, this phenomenon has been noticed by researchers – initially in America, where some radical changes at universities could be observed the earliest (McCabe et al., 2001, p. 220–221).

Publications analysing plagiarism have applied the normalising concept of “dishonesty” (*academic/student cheating/dishonesty/ fraud/misconduct*). In this approach, which was labelled as moral by Lee Adam, Vivienne Anderson and Rachel Spronken-Smith (2017, p. 19; cf. Blum, 2009, p. 149; Makridis & Englander, 2020; Shafaei et al., 2016), students are the guilty party who have to be caught in the act and effectively punished, with the punishment matching the severity of the crime. The language of legal and criminal proceedings is used, together with the notions of immorality and dishonesty. It is assumed that plagiarism is intentional. The second approach, called *regulative*, focuses on breaking university regulations as well as copyright-related ones. It is accepted that plagiarism can be unintentional. The most important objective is the formulation of university policies with a view to preventing this phenomenon and minimizing its scale. The language used refers to principles, guidelines and university traditions (Adam et al., 2017, p. 19; cf. Blum, 2009, p. 149). This approach also encompasses research focusing on the so-called academic integrity. Both these approaches are quite frequently interwoven in the literature (e.g. Foltýnek & Glendinning, 2015, 2015; Lancaster & Clarke, 2012).

Student culture of copying is studied predominantly through anonymous auditorium or Internet surveys to determine the scale of the phenomenon and attempt to discover its correlates. Both cheating and plagiarism have been the subject for research. The current knowledge reveals that the explanatory variables can be both individual features (gender, age, grade average, labour market status, income etc.) and group features (socio-cultural ones: field of study, peer pressure, school regulations including codes of ethics, copyright regulations, social norms defining (dis)honesty of specific behaviours, the structure of the higher education system, etc.). Numerous authors emphasize the significance of socio-cultural factors (cf. e.g. Crocker & Shaw, 2002, p. 40; Hattingh et al., 2020, p. 174; Lucas & Friedrich, 2005, p. 15; McCabe et al., 2001). Copying during university years is also researched as an independent variable – as a predictor of observing or breaking the formal rules in the world of employment (Lucas & Friedrich, 2005; Winrow, 2015).

The third approach described by Adam, Anderson and Spronken-Smith (*academic writing*) focuses mostly on unintentional plagiarism and assumes that plagiarism is an element of the learning process. It problematizes the concept of plagiarism, pointing out that it is impossible to create one interdisciplinary definition of such behaviours (Adam et al., 2017, p. 19; Angélil-Carter, 2014; Blum, 2009, p. 165–167; Chien, 2017, p. 118; Pabian, 2015). This approach also involves analyses of the so-called “patchwriting”, which means close connection to the paraphrased text, with only slight changes in wording or grammar. Such method of writing is considered to be a natural stage of learning academic writing and joining the academic community (Blum, 2009, p. 150; Howard, 1992, p. 233, 238). The concepts used to describe this phenomenon also include “intertextuality” and “grey zones”, i.e. the areas between what the evaluator considers to be legal / illegal, and what is efficient / inefficient from the point of view of the author of the text (Crocker & Shaw, 2002, p. 52–53). The academic writing approach discusses the phenomenon of unintentional plagiarism and uses the concept of copying (Doró, 2018, p. 3). Central and Eastern Europe is described here as an area where opportunities to learn academic writing during studies are scarce (Doroholschi et al., 2018, p. 6–8). Changes in this sphere are fostered by internationalization of education and introduction of the Anglo-Saxon model of writing (Angélil-Carter, 2014, p. 21; Crocker & Shaw, 2002, p. 41; Scollon, 1995). The related research uses qualitative methods much more frequently than in the case of the moral or regulative approach.

Polish research on student culture of copying has produced a significant number of publications focusing on the phenomenon of plagiarism (Bielska, 2015; Glendinning, 2015; Kowalski, 2017; Kozielski et al., 2017; Łozińska, 2018, p. 189–194; Mahmud et al., 2019; Sokołowska, 2020). Active contributors in this field are not only scientists but also providers of plagiarism detection systems (Kawczyński, 2007) and public institutions (the Polish Accreditation Committee and the Supreme Audit Office). An issue of importance is also the universal rule that students’ theses have to be tested by means of plagiarism detection systems; this also involved establishing a national repository of theses and obliged universities to perform such tests using the central public plagiarism detection system. Much less attention is given to cheating (Bielska & Hoffman, 2013; Gromkowska-Melosik, 2007), which is usually analysed with regard to lower levels of education (Kobierski, 2006).

Nowadays, researchers' focus has shifted in two directions worldwide. Firstly, the studies clearly concentrate on written works, where it is not only possible to detect potential plagiarism automatically (Do Ba et al., 2016; Foltýnek et al., 2019; Lancaster & Clarke, 2012) but also evaluate to what extent the work as a whole has been prepared by someone else with the consent of the original author (*contract cheating, ghostwriting, essay mills*) (Awdry & Newton, 2019; Bielska, 2015; cf. the opinion on the role of writing centres in Clarke, 1999). Secondly, a few researchers try to abandon the normative and regulative approach and adopt less judgmental notions and approaches, such as copying (Pabian, 2015) or learning (Adam et al., 2017).

We have decided to adopt the concept of *student culture of copying*, which allows a more distanced and non-judgmental (and thus more reliable) analysis. We take unintentional copying into consideration, but we also accept the interpretation that presents copying as a form of resistance against the requirements of the educational system (Blum, 2009, p. 148). We consider the analysis presented in this article to be located *between the academic writing approach and the regulatory approach*.

We analyse the phenomenon in the context of Poland as a semi-periphery country in the capitalist world system (Wallerstein, 2004) adapting to the models of the cultural centre. Poland is opening to the processes of internationalization of higher education and science; however, very few foreign students come to study at Polish universities, and there are also few foreign lecturers (Łuczaj et al., 2020; Rozmus & Kurek-Ochmańska, 2015). The stronger the interconnection of the Polish higher education system with other systems, the more frequently we are likely to observe the phenomena present in other systems (the US, Australia, the UK), including those regarding international plagiarism.

## Research Methodology

The three main research questions we intend to answer are as follows:

1. How do students understand plagiarism?
2. How do they describe their actual and possible behaviours in the situations in which they could plagiarize (working under time constraints, using foreign-language sources, using Internet sources)?
3. What is the context of plagiarism in diploma theses?

The study used the survey method with two techniques: auditorium survey and a questionnaire filled individually by respondents (both in Polish). The original goal of the study was to test the research techniques and their suitability for a large-scale representative survey. Here the data are analysed together. None of the respondents was tested twice; thus, we decided to combine the data. All analyses were conducted separately for each sample (auditorium vs. individual), and then for the combined sample. There are no significant differences in the results between them, so we decided to present the results for the combined sample. We have also decided to present the combined results from two surveys because if considered separately, each of them would be too small for drawing broader conclusions. In this sense, due to the variables studied, the survey results described here are *not representative* for the population of students. Our findings do not allow direct conclusions to be drawn about the students of the university under study nor for all the students in Poland. We do, however, offer possible explanations.

Besides the studies by other authors, the discussion of the results will refer to the findings from a study conducted in 2013. The main goal of the earlier research was to determine the scale of cheating and plagiarism and to learn the opinions held on this issue by students and teachers. Apart from an auditorium survey, the 2013 study also included individual in-depth interviews with university employees responsible for preventing and reacting to situations that were part of student culture of copying. Auditorium surveys were conducted during classes for first year Master program students (only full-time students), only one campus was included in the research.

The research tool used in this study was modelled on the earlier project. The tool used in 2019 was also partially modified to contain issues addressed in other studies (e.g. McCabe et al., 2001). The entire research tool consisted of 36 questions, including an open one, and a space for comments. The content of the questionnaire was exactly the same in both versions of the survey. At the beginning of the survey the respondents were informed that the participation is voluntary (informed consent).

The questionnaire was completed by 265 respondents. The study was conducted from March to June 2019. The subjects were solely Polish students of Bachelor or Master level studies (the latter group included both those who continued studies directly after their BA degree and those who returned for a complementary Master degree). The research tool was prepared in Polish. The study was conducted at two campuses of one university, which made it possible to survey different fields of education (social sciences, humanities, medicine, science etc.). The research project was accepted by the relevant Ethics Committee of the Faculty of Philosophy and Social Sciences of the Nicoluas Copernicus University in Torun. Ethical review was conducted and approved.

The pollsters'^3^ work was checked in June and July 2019. There was a place at the end of the questionnaire to leave contact details, which was optional. 182 respondents (69%) left their contact details. Following the fieldwork, a person who was not part of the pollsters team (the first author of this article) contacted all of these respondents by phone (text-messages) or e-mail and verified the question about their mother's education. The aim was to check that the pollsters did not falsify the questionnaires. No irregularities were discovered. The gathered data were also checked for their compliance with the paper version and tested for inconsistencies.

The data was analysed using IBM SPSS Statistics (version 26) and Microsoft Excel software. SPSS was used for cleaning the data and then for creating frequency and contingency tables. The question about the definition of plagiarism was an open one and was analysed in Excel. First, the data was read and the first list of recurring topics was identified. Second, the answers were coded (ascribed to one or more topics). Third, the list of topics was modified to include the whole spectrum of answers. Fourth, the answers were coded one more time.

Cheating and plagiarism always occur in the context in which the teacher is involved and the students’ behaviour depends on the teacher’s behaviour. However, since the study was conducted with students, the article is written mainly from their perspective. Data from teachers were not gathered.

## Findings

The sample included students between 19 and 50 years of age, from every university faculty. The majority of the respondents (89%) were within the age brackets associated in Poland with studying (19–24 years). The majority of them (77%) considered the financial situation of their families as good. Other socio-demographic features are presented in Table 1.

**Table 1:** Collected characteristics of the study sample (N = 265)

Gender		Female	168		63.4%	
		Male	92		34.7%	
		Other	2		0.8%	
		No data	3		1.1%	
Level of studies		Bachelor	172		64.9%	
		Master complementary	55		20.8%	
		Master continuation (post-BA)	34		12.8%	
		No data	4		1.5%	
Current employment status		Working	83		31.3%	
		Not working	182		68.7%	
Mother’s education (M)	Father’s education (O)		M	O	M	O
		Primary / unfinished primary school	10	4	3.8%	1.5%
		Vocational	67	109	25.3%	41.1%
		Secondary school graduate / post-secondary	89	63	33.6%	23.8%
		Bachelor / Engineer degree	18	15	6.8%	5.7%
		Master, PhD or post-graduate	73	59	27.5%	22.3%
		Unknown	7	14	2.6%	5.3%
		No data	1	1	0.4%	0.4%

Source: Authors’ own work

### Interpretation of the Norm: Definition of Plagiarism

In the learning process, copying (including plagiarism) can be considered as a step towards mastering academic writing skills. However, to take this step, students must formulate their own definition of what is allowed and what is not. A question arises as to how they understand the norms regarding the acceptability of copying. In order to answer the first research question, we asked the respondents how they understood plagiarism.^4^ It was an open question. Five recurring elements of the definition could be identified.

Claiming the ownership for somebody else’s authorship is a definition which occurred most frequently (216 answers, 82%). Typical descriptions were phrased as follows.

*Using content produced by others and presenting it as one’s own.* (70).^5^

The second element most frequently mentioned in respondents’ definitions of plagiarism was a reference to the form of the text/work (192 answers, 73%).

*In my opinion, plagiarism means copying another author’s text word for word and claiming the authorship.* (113).

Copying, also word for word, is the next characteristic students took notice of (112 answers, 42%) in the context of defining plagiarism.

*Copying of the content that comes from someone else; as above, word-for-word copying.* (11).

Lack of references to sources (96 answers, 36%) is the fourth element of the definitions.

*Plagiarism is using somebody else’s work without acknowledging the source, and ascribing the authorship to oneself.* (101).

The last element of plagiarism can be described as its moral assessment. Part of the respondents (21 persons, 27%) considered this process as negative, using such terms as “stealing”, “appropriation”, or “cheating”. Additionally, the respondents pointed out that for plagiarism to occur, the person committing it must be aware of the fact, do it on purpose and for their own benefit.

*Stealing of intellectual property.* (23).

In the respondents’ definitions, fraud is associated with the deterioration of the victim’s property rights. In the context of plagiarism, it means more than the economic consequences: it also involves respect and prestige. Interestingly, there was also an answer that presented the opposite situation in the context of fraud – that plagiarism involves cheating not others, but oneself; it means working against one’s own convictions and objectives.

*[It is] cheating against oneself.* (120).

The listed definitions of plagiarism lead to the conclusion that respondents consider authorship to be a specific, legally protected form of relations between the author and the user of a given work (but not a broader community). Such a relation requires appropriate treatment of the creator (marking their input, taking care of their well-being and profits) and the work (conscious use of appropriately sized fragments, correct bibliographical annotation, skilful paraphrasing and quoting). A work is understood predominantly as something that has a written form/is a text.

It is also evident that the process of learning how to write (moving from lack of understanding of the non-plagiarism norm to comprehending it) must necessarily include some “in-between” areas (on “grey zones”, see Crocker & Shaw, 2002, p. 52–53), some intermediary phases of learning, which involve making mistakes. Such errors can be identified in three spheres of the quoted definitions: the authorship, the object and the writing process. Regarding authorship, there appeared an incorrect conviction that a work can be used only with the author’s permission or knowledge (21 persons, 8%) and that plagiarism occurs only if the quoted texts have been published before (two persons, 1%). It should be noted that students write their texts for educational purposes and use primarily the right to quote – thus the author’s permission or knowledge are not required. It concerns not only written work but also graphic sources, provided that the purpose of the citation is explanation, polemic, critical or scientific analysis. In addition, the person who is quoting is obliged to indicate the source and the creator of the quoted graphics (Machała, 2020 p. 82–86). This is specific to the Polish context and is differently regulated in other countries.

The respondents described the objects of plagiarism in a variety of ways. Misunderstanding the norm is particularly evident when enigmatic words are used, such as “content”, “information”, “sources”, “fragments” or even the unspecified “something” (26 persons, 10%). The intermediate forms are the mentioned reductions of the plagiarism definition to text forms/”written works” only (194 persons, 73%). Clear understanding of the norm can be noticed in those answers which 1) spoke about various forms of creativity (“output”, “work”, “piece”, “author’s work” etc.; 40 persons, 15%); 2) gave examples other than (written) text (e.g. “paintings” or “music”; 45 persons, 17%); 3) referred to the precisely defined but broad phrase – “intellectual property” (22 persons, 8%).

In case of the writing process, one extreme is the most common understanding of plagiarism only in the categories of copying word for word, as described above (112 persons, 42%). An intermediate form is the accepted copying of entire works (24 persons, 9%). Part of these definitions would include contract cheating. Other intermediate forms included enumeration of plagiarism types (e.g. partial, full and hidden), as well as defining the scope of changes – the students reported that nowadays it is not enough to have a “slightly altered” text, introduce “minor changes” or “change the word order” to avoid plagiarism; plagiarism occurs when we observe “evident use”, “in a significant quantity”, or “lifted without changing”.^6^ Such additional information could be found in the answers of 19 persons (7%). The most competent respondents pointed out that plagiarism can also apply to paraphrasing (“in one’s own words”, 3 persons, 1%).

Several persons mentioned they were unsure how much a text should be changed (“I think”). This lack of certainty was also clear in the optional comments added at the end of the questionnaire, to which we will refer further.

Most of the definitions are very brief, but they lead to a conclusion that, in principle, the respondents know what plagiarism is, at least in its most extreme form (ascribing the authorship of a text to oneself by word-for-word copying without adding a reference). Students’ definition of plagiarism are diverse, with imprecise definitions prevailing, but the most extreme form of plagiarism is understood correctly.

Hence the social norm of non-plagiarism was accepted by our research subjects, at least declaratively, but not necessarily internalized. More complicated situations would certainly pose difficulties. Thus, we decided to include questions regarding hypothetical situations.

### Redefinition of the Norm in Hypothetical Situations—Omitting the Norm

**Chart 1:**
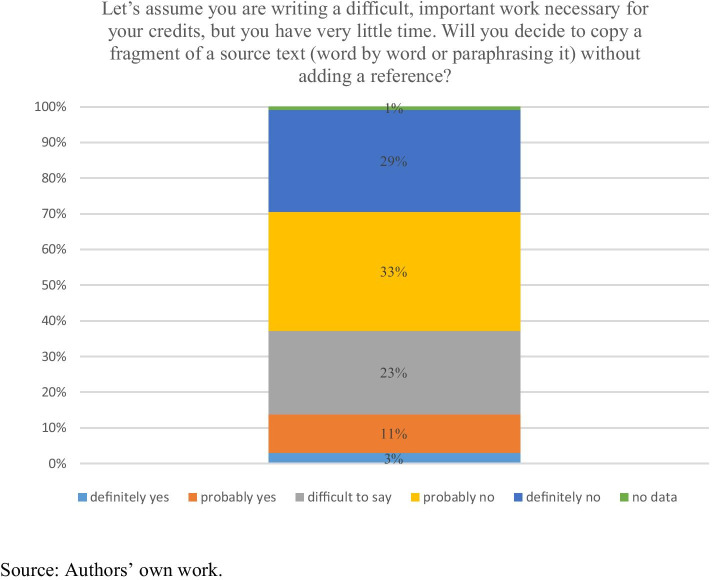
Dilemma No. 1 – Time constraints. N = 265

**Chart 2:**
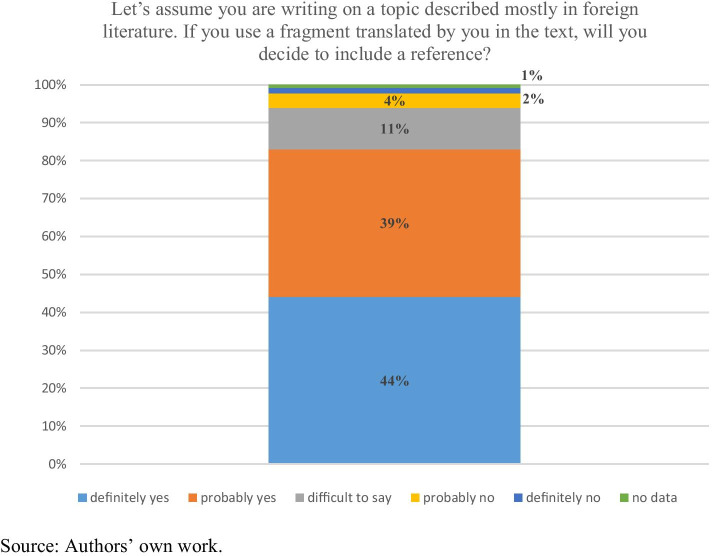
Dilemma No. 2 – Use of foreign literature. N = 265

In our research tool we included a series of hypothetical questions to find out in more detail how the students deal with dilemmas common in the process of studying. In order to answer the second research question, we asked them to estimate how they would behave in three situations: when working under time constraints, when using foreign-language sources and when using Internet sources. The questions were intentionally constructed so that they would represent the way the respondents perceive the non-plagiarism norm rather than their actual behaviours. It should be noted, however, that questions presented in Chart 1 and 2 are leading questions (students knew the “right” answer). Thus, they should be interpreted with caution.

Limited time (Chart 1) is a significant argument in favour of committing plagiarism for 14% of the respondents. Interesting is the group of indecisive people, comprising as many as a quarter of the respondents (23%), which confirms that copying is a more or less effective strategy in specific situations (cf. Crocker & Shaw, 2002, p. 52–53). Two thirds of the respondents would not decide to copy word for word without a reference – a result consistent with the plagiarism definitions presented above. Such extreme plagiarism is rather well recognised.

The second dilemma concerned foreign languages sources. Among the respondents, 83% would include a reference if the fragment they translated and included in their work came from foreign literature. Only one in ten is undecided. As few as six out of a hundred would commit plagiarism. It should be noted that for a large share of the respondents such situation may be extremely hypothetical as they hardly ever use foreign-language sources – they study in Polish.

**Chart 3:**
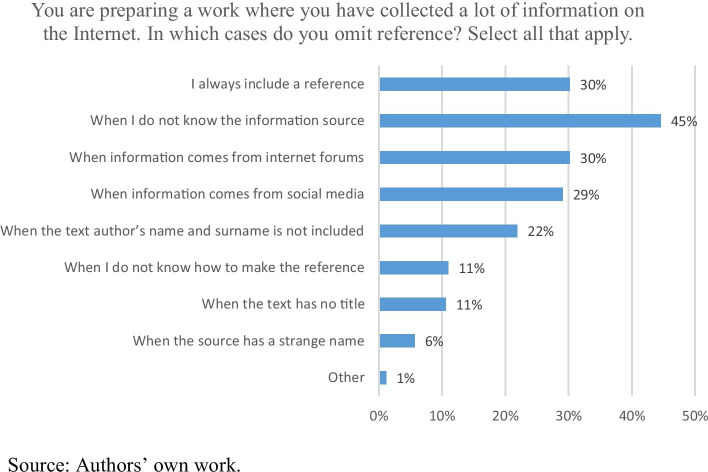
Dilemma No. 3 – Internet sources. N = 265

More detailed information on students’ attitude towards Internet content is provided by the question regarding the third hypothetical situation (Chart 3). Only 30% of the respondents declare they always provide a reference, even if it is difficult for them. The rest try to find their way around the missing data (origin of information, author, title) and their own lack of competences (unusual Internet sources, strange names, lack of relevant skills). What causes most problems are sources of unknown origin, i.e. found by accident or during other Internet activities. It can also be noted that students have problems with referencing social media and Internet forums, i.e. sources which are not typical Internet articles so the majority of guidelines (older editions in particular) may not include instructions on how to cite them. It is evident here that the Internet contains many sources of varying quality, difficult to reference for the majority of the respondents. When they lack information or skills, they choose not to provide a reference, and sometimes not to use the source at all. The latter situation may have both benefits (omitting sources of doubtful quality) and drawbacks (systematic overlooking of valuable sources).

In the situations involving either time pressure or using foreign literature, the majority of the respondents declare complying with the non-plagiarism norm. These answers reflect the convictions of over 70% students who consider plagiarism to be a negative phenomenon. Only 5% express the opposite view, while one-fifth have no opinion on this topic. Similar indecisiveness was noted with regard to using Internet sources, and was visible when we attempted to estimate the scale of the problem. More than half of the respondents (52%) “find it difficult to say” whether plagiarism is a common phenomenon, 20% consider it to be common, and 17% disagree. The difficulty in answering this question may result from the fact that plagiarism is relatively difficult to discover. Thus, based on the results, we can argue that the non-plagiarism norm is imprecise and in complicated situations students try to *omit it* (*they withdraw from using a source in fear of making a mistake*). Such a norm may be prone to redefining – when it is not clear how to act, one must invent a way. This applies both to students and academic teachers.

### Redefinition of the Norm in the Learning/Teaching Process – Limiting the Norm

If plagiarism is considered a natural mistake made by students during the process of learning how to write academic texts, it seems worthwhile to determine whether the respondents have been prepared for such tasks, whether their curricula included classes devoted to this subject matter, and whether their diploma projects supervisors paid special attention to it. These issues are covered by our third research question.

More than a third of the respondents (36%) declare that they have not been taught how to reference citations correctly; 8% do not know how to do it or are not able to recall it. More than half (55%) have had the opportunity to learn such skills and – as we have demonstrated above – have acquired some basic knowledge and are able to pinpoint extreme forms of plagiarism. When we filter the received answers with the Bachelor/Master level used as a variable, it becomes evident that it is the study level that makes the greatest difference. As few as 18% of second-level students declare that they have never learnt how to make correct references, while 80% have such knowledge. Only 38% of first-level students have been instructed on the citation rules (50% have not, 9% do not remember^7^). This discrepancy may result from the experience of writing the Bachelor’s thesis. We suppose that another reason for such a discrepancy may be the fact that not in all fields of study in Poland, BA or MA seminars start in the first year of study.

In the case of the first-level students, there have either been no classes on this topic, or, even if there have, they have not included practice. Such hypothesis is confirmed by the respondents’ comments added as observations and conclusions at the end of the questionnaire, an optional part to complete.

The respondents wanted to make use of this opportunity to, paradoxically, emphasize their lack of knowledge. They were not sure what exactly plagiarism is as they had not been taught it or the classes came too late.

*At university there are no classes about the rules of correct citation and the thesis form – diploma supervisors often are unable to help students.* (225).

In this context students try to redefine the non-plagiarism norm to some extent. The main change is to divide works into the less important (to obtain credits) and the more important (diploma projects). The fact that a larger percentage of respondents declare committing plagiarism in a work written for a credit than in a diploma project seems to confirm our conclusion. Two-fifths of the respondents (40%) declared that during their studies they did copy a fragment of a text from a source without referencing it in their credit-level work. As other sources confirm, plagiarism detection rate is negligible. While 105 people declared having committed plagiarism, the teacher noticed it in ten cases only. The issue was usually pointed out and the students were expected to revise their work. More strict measures such as a request to write a new text on another topic, a lowered grade or a disciplinary talk with the teacher were used in very few cases. One person directly declared that although their plagiarism was discovered, they did not face any consequences. As regards diploma projects, the percentage of committed plagiarism is significantly lower – 10%.^8^ Supervisors noticed plagiarism only in four cases (out of seventeen). As in the case of the less important works, the students were reprimanded and obliged to revise their theses. One person noted that they had a disciplinary talk with the supervisor.

Plagiarism detection rate is much higher for theses than for credit-level works although more people commit it in the latter case. The first reason is that there are usually more students writing credit-required works than those attending a BA/MA seminar. Secondly, diploma projects are considered by students and supervisors to be more significant as plagiarism would entail more serious consequences (stripping of the academic title).

We also asked the students whether they would consider an offer to present somebody else’s work under their own name, by which we meant a situation when a third party offers to write (partially or fully) a credit-level work or a diploma project for them. As we could have expected, there was more hesitancy in the case of diploma projects, where such an offer would be accepted by 10% of the respondents and rejected by 59%; in the case of credit-level work these numbers were 22% and 79% respectively. We could conclude that the second strategy students apply is *reducing the scope of the norm applicability (limiting the norm).* They treat plagiarism in diploma theses more seriously.

## Discussion

If we compare the definitions of plagiarism presented by students in our 2019 study to the data from the 2013 study, the results did not change. The above categories are quite similar to the set identified in the earlier project. Students understand verbatim copying (Walker, 1998, p. 103), have problems with other types of plagiarism and suggest (in comments) that they were not prepared to cite sources correctly. The previous research (2013) confirms that the thesis supervisors are supposed to teach such skills only in seminar classes (Bielska & Hoffman, 2013, p. 64-65).

The report of Supreme Audit Office (Najwyższa Izba Kontroli [NIK]) in Poland states that 95% of students were informed how they should cite literature and source materials. They did, however, emphasize that this knowledge was theoretical and not at all, a little or moderately useful (NIK, 2014, p. 27). In the research project comparing the UK, Poland, Romania and the Czech Republic, only 15% of Polish students declared that they “have received training in techniques for scholarly academic writing and anti-plagiarism issues” (Mahmud et al., 2019, p. 280). Limited knowledge of students was described also e.g. by Shih-Chieh Chien. A study of 60 Taiwanese students demonstrated that the majority of them had some knowledge about plagiarism, but during exercises in writing they were usually unable to recognize actual cases of plagiarism (Chien, 2017, p. 118). Another study conducted at a big private university in the USA presents similar conclusions: some of the participants believe that the vague and non-explicit definitions provided by instructors leave “*grey areas*” in the understanding of what it means to cheat (Wei et al., 2014, p. 293). This conclusion is also confirmed by Anna Sokołowska’s research study (2020, p. 222–223) conducted in Poland.

The comparison of the results presented in Chart 1 to the 2013 survey shows that the proportions of answers in the previous study were slightly different: the respondents much more frequently declared obeying the non-plagiarism norm (78% vs. 62%) (Bielska & Hoffman, 2013, p. 34). Age can offer a kind of explanation here: the younger the students are, the more contact they have had with new IT technologies where authorship is less important than content sharing (Blum, 2009, p. 167). Also, printed and non-printed (Internet) content may have a different status in the eyes of the respondents – non-printed sources are more likely to be unconsciously perceived as belonging to the public domain, i.e. not requiring a reference (Baruchson-Arbib et al., 2004, p. 4). Similar issues were researched by Polon Šprajc, Marko Urh, Janja Jerebic and their colleagues. The study was conducted at a university in Slovenia with the sample of 139 students; the method used was questionnaire (Šprajc et al., 2017, p. 30). Among the reasons for plagiarism in the group of information and communication technologies (ICT and the Internet) the most important were: “Thanks to modern technology it is easier to copy/paste” and “I can easily access Internet materials” (Šprajc et al., 2017, p. 35–37).

The comparison of the results presented in Chart 2 to the 2013 survey shows that in 2013 the respondents declared similar acceptance of the non-plagiarism norm from foreign literature: 89% of them would include a reference (Bielska & Hoffman, 2013, p. 35; cf. Crocker & Shaw, 2002, p. 46). As we showed (Chart 3) using Internet sources is more difficult for students (see also Sokołowska, 2020, p. 223–224). Similar conclusions were drawn by Lea Calvert Evering and Gary Moorman. In their view (2012, p. 37), one of the most common reasons for plagiarism is the growing diversity of the digital media which students have everyday contact with, as well as the growing role of the Internet as an information source. Therefore, this situation raises a level of uncertainty as to whether information is used properly, especially since Internet resources – with or without a specific author – are accessible practically incessantly. Weber et al. (2018, p. 14–15) point out that it is difficult to teach students the computer skills that would help them search for digital information because such courses usually involve handing the students coursebooks and guidelines. As a result, after initial instruction many students return to their old habits, including doing simple Google searches. However, the Internet cannot be blamed for everything since plagiarism has been common among students at least since the 19^th^ c. (Simmons, 1999, p. 41–42).

Generally, the phenomenon of plagiarism applies not only to students but also to academic teachers (Farahian et al., 2020, p. 1). This issue has emerged in a study conducted in 2013 when the respondents wrote that “the example comes from the top”, so—as they believe—teachers cannot criticize them for doing this. However, in our survey, students did not mention (appropriate or inappropriate) examples set by teachers. Nevertheless, the 2013 study also showed that students treat plagiarism in diploma theses more seriously (Bielska & Hoffman, 2013, p. 49).

The reason that can help explain why at universities there is no particular focus on referencing can be related to the fact that in Poland for nearly 15 years the number of students per one academic teacher^9^ has significantly increased – from 14 to 20 students in public universities and from 25 to 40 students in private ones (GUS, 2014, p. 44–45; GUS, 2017, p. 44–45). Mass education,^10^ among other variables, leads to the situation where the lecturers and thesis supervisors face a difficult task: on the one hand, they must pass all the knowledge they have planned to, and on the other, they also have their research and organizational duties. It is well known that for many university employees research work is more important than teaching (Schmidt, 2017) as the former is the main factor deciding whether their contract with the university is prolonged. Thus, teaching students becomes a matter of secondary importance; after the introduction of the point-based system of evaluating scientific achievements, academic teachers focus more on their work on the “parametric game”^11^ (Kulczycki, 2017). These tensions – inherent in the role of an academic teacher and researcher (Kwiek, 2015, p. 22) as well as in the role of a student lacking appropriate training – lead both sides of this relationship to redefining the norms on copying to some extent.

## Conclusions

The analysis presented from this research helps to fill the gap in knowledge about higher education in Poland and fall within the trend of the academic writing approach in research into plagiarism, which is new to the Polish context. The collected data confirm that plagiarism derives from the complexity and ambiguity of the very concept of plagiarism. Compiling a correct reference can be a challenge even to experienced scientists; it is thus no wonder that it may be a daunting task for students. This phenomenon can be illustrated by the words from one of our respondents quoted in the title: “There must be someone’s name under every bit, even if it is not really important or even correct,” which clearly reveals not only lack of understanding of referencing rules but also the feeling that this aspect of learning is over-regulated. It could be interpreted as a form of *resistance*.

The surveyed students effectively accepted the non-plagiarism norm in reference to its extreme case, i.e. copying word for word without including a reference. This is confirmed by their personal definitions of plagiarism and by the declared behavioural choices in hypothetical situations – they say they will not commit such plagiarism either under time pressure nor when using foreign sources. Internet sources pose a much greater difficulty – both due to the variety of forms (scientific articles, press articles, blog entries, comments on forums etc.) and the lack of practical and useful guidelines^12^ on how to deal with different aspects of this diversity (how to reference an article and how to do that for a forum entry). If there is no precise information on the source and/or they lack proper competences, students decide not to make a reference. This is the first redefinition of the non-plagiarism norm we have identified: *withdrawing in fear of making a mistake (omitting the norm)*.

The second redefinition of the non-plagiarism norm identified by us is *reducing the scope of the norm applicability (limiting the norm)*: we can notice an emerging division into areas where the non-plagiarism norm must be observed more closely and those where it is not so important. This is connected with the penalties for breaking the norm. The respondents classify works as credit-level and diploma-level texts. Plagiarism is not allowed in the latter as it may mean being stripped of the academic degree. In the former case, one can do it sometimes – detection rate is poor and consequences are not severe. This redefinition is also reflected in the respondents’ definitions of plagiarism. Many of the definitions mentioned a “grey zone” (Crocker & Shaw, 2002, p. 52–53) – a space within which there could be a *space for learning and improving academic skills* while making mistakes as well as a space for *unintentional plagiarism*.

Our study confirms that socio-cultural factors are crucial to understanding the phenomenon of plagiarism, and the frame of academic writing approach is particularly useful in this regard. The presented conclusions are of particular importance for those in charge of planning syllabi and curricula, as well as for academic teachers in general. They demonstrate that even the strictest anti-plagiarism policies and plagiarism detection systems will not help to improve the students’ understanding of the ambiguous norm. It is the direct and close contact during the learning process that allows teachers to convey not what plagiarism is but how authorship is defined in a specific field at a given time, and what are the field-specific models of recognizing and ascribing authorship.

## Footnotes

1 Studying in Poland is in principle free of charge (no tuition fees); the majority of students learn at public schools which are considered to be more prestigious than private ones.

2 But are not limited to verbatim copying.

3 Hired researchers who conducted the study in the field, but did not take part in the process of designing methodology or analysing data.

4 14 questionnaires were discarded due to incomplete data.

5 It means the respondent's number in the SPSS database.

6 Words in quotation marks in this sentence are citations from respondents.

7 All discrepancies in the percentages (the total not equalling 100%) result from (minor) lacks of data.

8 For N = 180, 85 respondents marked the answer “not relevant, not writing my diploma project yet”.

9 At every university the actual student-to-academic teacher ratio depends mostly on classes that they teach for a particular faculty. A professor who gives a lecture on law where there are approximately 500 students in the audience is obliged to test them all, while in the philosophy department a teacher can have only five people attending classes. Thus, the above mentioned indicator should be studied with caution as it does not reflect the real situation at universities.

10 It should be stated that massification could not be presented as the only factor causing plagiarism. It is rather a catalyst for processes which had already existed.

11 The „ parametric game” refers to researchers who want to fit in the system of the evaluation of science in Poland. They apply two strategies: they publish only in high Impact Factor journals (a lot of points for one article which is hard to publish—“impactitis”) or they publish many papers in journals without Impact Factor (few points for one article which is easy to publish – “running for points”) (Kulczycki, 2017).

12 In Poland (NIK, 2014, p. 27–29; cf. writing centres in Clarke, 1999).
